# Direct clinician–pathologist multidisciplinary review and diagnostic revision in children with gastrointestinal disorders

**DOI:** 10.1002/jpr3.70239

**Published:** 2026-09-03

**Authors:** Emma Dahl, Christa Bergheim, Irina Berger, Ralph Melchior, Andreas Jenke

**Affiliations:** ^1^ Department of Pediatrics, Faculty of Health University of Witten/Herdecke Witten Germany; ^2^ Department of Neonatology and Paediatric Gastroenterology Children's Hospital Kassel Kassel Germany; ^3^ Department of Pathology Klinikum Kassel Kassel Germany

**Keywords:** endoscopy, histology, inflammatory bowel disease

## Abstract

Accurate pediatric gastrointestinal (GI) diagnosis often depends on integrating clinical, endoscopic, and histopathologic information. We evaluated the impact of weekly face‐to‐face multidisciplinary team (MDT) meetings on diagnosis in children undergoing endoscopy or liver biopsy at a tertiary center. This single‐center study included 53 patients discussed between March and November 2025; because 6 children were reviewed twice, the analysis was case‐based (59 MDT discussions). Diagnostic change was defined as any revision, reclassification, refinement, or exclusion of a suspected diagnosis after MDT review. Overall, diagnosis changed in 12 of 59 cases (20.3%). Endoscopic classifications changed significantly after MDT discussion (*p* = 0.0091), as did histopathology classifications (*p* = 0.0118). Pathologist experience was associated with pathology revision (*p* = 0.0293), whereas patient‐specific parameters and endoscopist experience were not. Endoscopy requests to pathology were frequently sparse. These findings suggest that regular clinician–pathologist MDT review can improve diagnostic precision in pediatric GI practice.

## Abstract (highlights)

What is Known

- Accurate diagnosis of pediatric gastrointestinal (GI) disorders requires integration of clinical, endoscopic, and histopathologic findings.
- Multidisciplinary review can improve clinicopathologic correlation, but evidence for routine clinician–pathologist multidisciplinary review in pediatric gastroenterology is limited.

What is New

- Weekly face‐to‐face multidisciplinary review was associated with diagnostic revision in 20.3% of pediatric GI cases discussed.
- Both endoscopic and histopathologic classifications changed significantly after multidisciplinary review, with pathology revision associated with pathologist experience.
- Endoscopy requests to pathology frequently lacked detailed clinical and endoscopic information, identifying a potentially modifiable communication gap.

## 1 INTRODUCTION

Accurate diagnosis in pediatric gastrointestinal (GI) disease depends on integrating clinical presentation, endoscopic appearance, and histopathology. This is particularly important in inflammatory bowel disease (IBD), eosinophilic esophagitis (EoE), celiac disease, and nonspecific inflammatory conditions, where isolated interpretation of endoscopic or histologic findings may be misleading.^1^, ^2^ Published pediatric guidance emphasizes systematic endoscopic sampling and clinicopathologic correlation.^1^, ^2^ Multidisciplinary team (MDT) review may therefore be especially useful when initial findings are equivocal, incomplete, or discordant. Team‐based care has been associated with improved care processes and patient experience in other settings, and systematic reviews of GI MDTs suggest that case discussion can alter diagnosis and management.^3^, ^4^ However, data on routine clinician–pathologist review in pediatric gastroenterology remain limited. We evaluated whether weekly face‐to‐face MDT meetings altered endoscopic or pathology‐based diagnoses in children undergoing GI investigation at a tertiary center.

## 2 METHODS

### 2.1 Ethics statement

This study was reviewed and approved by the Ethics Committee of the State Medical Association of Hessen (Ethik‐Kommission bei der Landesärztekammer Hessen), Germany, on July 1, 2025 (reference number: 2025‐4112‐evBO). Informed consent was waived by the Ethics Committee as the study utilized pre‐existing, de‐identified data.

### 2.2 Study design

We performed a single‐center study in children aged 0–18 years who underwent endoscopy or liver biopsy at Klinikum Kassel and were discussed in a weekly pediatric GI MDT between March 1 and November 30, 2025. Clinical data were extracted prospectively from the medical record after local approvals.

During the study period, 54 patients were discussed, representing 10.7% of all eligible patients. One patient was excluded because of age, leaving 53 patients for analysis. As six children were discussed on two separate occasions, the analysis was performed on a case‐discussion basis, comprising 59 MDT case discussions in total. The decision to present a patient at the MDT was made by the consultant pediatric gastroenterologist responsible for the patient's care, particularly if histopathological findings were unsuspected and did not correlate with the clinical diagnosis.

For each case, the pre‐MDT endoscopic impression and histopathology diagnosis were compared with the post‐MDT conclusion. A diagnostic change was defined as any revision, reclassification, refinement, or exclusion of a previously suspected condition after discussion. We also recorded patient age and sex, operator and pathologist experience, and the content of endoscopy requests submitted to pathology.

### 2.3 Statistical analysis

Associations were tested with Fisher's exact tests because of small cell sizes; *p* < 0.05 was considered significant.

## 3 RESULTS

The 53 included children contributed 59 case discussions. Mean age at the time of procedure was 10.5 years, and 62.3% were male. Most endoscopies were performed by senior registrars (72.9%), while pathology reports were most commonly signed by a consultant together with a registrar (44.1%) or by a senior registrar alone (25.4%) (Table 1).

**Table 1:** Cohort and reporting characteristics.

Characteristic	Category	Value
Study sample		
Patients included	—	53
MDT case discussions	—	59
Age, years	Range	0.008–18.0
	Mean	10.9
Sex	Male	18/53 (34.0)
	Female	35/53 (66.0)
Clinician experience at MDT discussion (*n* = 59)		
Clinician experience	Consultant	9/59 (15.3)
	Senior registrar	43/59 (72.9)
	Consultant/senior registrar	6/59 (10.2)
	Senior registrar/core trainee	1/59 (1.7)
Pathologist experience at MDT discussion (*n* = 59)		
Pathologist experience	Consultant	10/59 (16.9)
	Consultant/senior registrar	26/59 (44.1)
	Consultant/specialist registrar	4/59 (6.8)
	Consultant/core trainee	4/59 (6.8)
	Senior registrar	15/59 (25.4)

*Note*: Values are shown as *n*/*N* (%) unless otherwise stated. Patient‐level variables use *n* = 53; MDT case‐discussion variables use *n* = 59.

Abbreviation: MDT, multidisciplinary team.

Across all case discussions, the MDT led to diagnostic revision in 12 of 59 cases (20.3%) for both endoscopy‐based and histopathology‐based diagnoses. Endoscopic classifications changed significantly after MDT review (*p* = 0.0091), and pathology classifications also changed significantly (*p* = 0.0118) (Table 2). On the endoscopy side, revisions most often involved Crohn disease, celiac disease, and cases initially labeled as unspecified inflammation or IBD‐unclassified. Specifically, two cases initially diagnosed as non‐specific duodenal inflammation were reclassified as celiac disease; in one case, *Helicobacter pylori* organisms were identified; and in one case, reassessment of eosinophil counts together with basal cell hyperplasia in the esophagus supported a diagnosis of eosinophilic oesophagitis. The remaining eight cases involved IBD‐unclassified, Crohn's disease, ulcerative colitis, and infectious colitis, with diagnoses revised after detailed multidisciplinary discussion incorporating all available clinical, endoscopic, histological, and laboratory parameters. After discussion, endoscopic impressions were commonly reclassified to unspecified inflammation, Crohn disease, infectious colitis, or exclusion of celiac disease. On the pathology side, the most frequent post‐MDT reclassification was to Crohn's disease.

**Table 2:** Key outcomes of MDT review.

Outcome/variable	Value, *n*/*N* (%)	*p* value
Diagnostic changes after MDT review (*n* = 59)		
Endoscopic classification changed after MDT review	12/59 (20.3)	0.0091
Histopathological classification changed after MDT review	12/59 (20.3)	0.0118
Factors associated with histopathological revision		
Pathologist experience	—	0.0293
Primary reason for diagnostic revision (*n* = 12)		
Resolution of borderline or equivocal findings	3/12 (25.0)	—
Integration of clinical, laboratory, radiological, endoscopic, and histopathological data	8/12 (66.7)	—
Additional focused review of the histological specimen	1/12 (8.3)	—
Quality of information provided in pathology requests		
Request limited to 1–2 words	21 (39%)	—
Clinical symptoms not documented in the request	17 (33.9%)	—
Visible endoscopic findings not documented in the request	20 (37%)	—

*Note*: Values are shown as *n*/*N* (%) where denominators are explicit; otherwise, counts are shown as provided.

Abbreviation: MDT, multidisciplinary team.

There was no significant association between patient‐specific parameters and diagnostic change. Likewise, endoscopist experience was not associated with revision. In contrast, pathologist experience was significantly associated with pathology diagnosis change (*p* = 0.0293), with revisions occurring most often in cases initially reported jointly by consultants and senior registrars (Table 2).

Review of endoscopy requests submitted to pathology showed variable quality. A stated diagnosis was present in 83.1% of reports, but many requests were very brief, and 39.0% consisted of only one or two words. Notably, among the 12 cases requiring diagnostic revision, only two were accompanied by such brief endoscopic requests. In addition, specific clinical symptoms were not documented in 33.9% and visible endoscopic findings in 37% (Table 2).

## 4 DISCUSSION

In this study, one in five pediatric GI cases discussed at MDT underwent diagnostic revision, and both endoscopic and histopathologic classifications changed significantly after face‐to‐face multidisciplinary review. The findings support the idea that direct clinicopathologic discussion adds value in pediatric gastroenterology, especially in diagnostically difficult entities such as pediatric IBD, EoE, celiac disease, and nonspecific inflammatory presentations, where diagnosis often depends on combining morphologic findings with clinical context and sampling information.^1^, ^2^, ^5^, ^6^ Overall, the results demonstrate the added value of multidisciplinary expertise and an integrated diagnostic approach. As such structures are most commonly available in tertiary or specialized centers, the findings also underline the importance of referral pathways for children with rare, complex, or diagnostically uncertain GI and hepatological disorders. Access to specialized multidisciplinary review may therefore contribute to more accurate diagnosis, improved disease classification, and more appropriate subsequent management.

Our observations are consistent with the recent adult IBD study by Kim et al., in which 15%–42% of cases discussed at a multidisciplinary conference had a diagnostic change across the first three reviews, and most changes were attributed to expert pathology reinterpretation.^5^ That study also showed that diagnostic revision could lead to changes in medication, surgery, and short‐term clinical improvement, reinforcing the idea that expert pathology input does not merely relabel disease but can change management. Although our pediatric cohort was smaller, case‐based, and included a broader spectrum of GI indications, the direction of effect was similar: structured discussion with pathology review refined diagnosis in a clinically meaningful proportion of children. The association between revision and pathologist experience in our data further suggests that MDT review may be especially valuable when cases are interpreted across training levels or when subspecialty review is incorporated into the conference.

The descriptive analysis of endoscopy requests also points to a modifiable systems issue: Pathology interpretation is being asked to answer complex clinical questions despite limited information in the initial requisition. Improving the quality of requests may enhance first‐pass diagnostic accuracy and reduce later revision. This study has limitations. It was retrospective, single‐center, and relatively small, and no control group without MDT discussion was available. Some children were discussed more than once, and we could not separate the independent effects of additional clinical information, endoscopic reinterpretation, and histologic re‐review. The findings therefore reflect real‐world service impact rather than causal proof. Nevertheless, the observed diagnostic revision rate suggests that regular pediatric GI MDT meetings can meaningfully refine diagnosis and may also offer an educational benefit for trainees.^4^, ^7^

## 5 CONCLUSION

Weekly pediatric GI MDT meetings were associated with significant revision of both endoscopic and histopathologic diagnoses, with an overall change rate of 20.3%. Variability in endoscopy request quality and the association with pathologist experience suggest that both structured multidisciplinary review and better front‐end clinical information may improve diagnostic precision in children with GI disease.

## CONFLICT OF INTEREST STATEMENT

The authors declare no conflicts of interest.

## Data Availability

The data that support the findings of this study are available on request from the corresponding author. The data are not publicly available due to privacy or ethical restrictions.
